# 非小细胞肺癌腹膜转移预后因素的单中心回顾性分析

**DOI:** 10.3779/j.issn.1009-3419.2019.03.04

**Published:** 2019-03-20

**Authors:** 宝山 曹, 燕娥 刘, 文琤 尹, 倩 李, 莉 梁

**Affiliations:** 100191 北京，北京大学第三医院肿瘤化疗与放射病科 Department of Medical Oncology and Radiation Sickness, Peking University Third Hospital, Beijing 100191, China

**Keywords:** 肺肿瘤, 腹膜转移, 总生存期, Lung neoplasms, Peritoneal carcinomatosis, Overall survival

## Abstract

**背景与目的:**

腹膜转移（peritoneal carcinomatosis, PC）在肺癌中较为罕见，但预后极差，目前影响PC发生和预后的因素尚不清楚。本研究旨在探讨非小细胞肺癌（non-small cell lung cancer, NSCLC）PC的临床病理特征和治疗情况对预后的影响。

**方法:**

回顾性分析2010年8月-2018年8月于北京大学第三医院肿瘤化疗与放射病科接受治疗的NSCLC、符合入组条件的PC患者，采集年龄、性别、胸腔积液、基因状态等资料，应用*Kaplan-Meier*方法进行生存分析。

**结果:**

共12例PC患者入组，均为异时性转移，PC发生率为1.44%（12/836）；12例患者均为腺癌，确诊肺癌时50%（6/12）患者合并胸腔积液，确诊PC时100%（12/12）患者合并胸腔积液；12例患者中9例含有表皮生长因子受体（epidermal growth factor receptor, *EGFR*）、间变性淋巴瘤激酶（anaplastic lymphoma kinase, *ALK*）、*ROS*原癌基因1受体酪氨酸激酶（ROS proto-oncogene 1 receptor tyrosine kinase, *ROS1*）突变；含有基因突变患者一线治疗开始后的中位生存期（median overall survival 1, mOS1）和确诊PC后的中位生存期（medial overall survival 2, mOS2）显著高于无突变组，分别为26.0个月和6.0个月比10.0个月和1.5个月（*P* < 0.05）；确诊PC后治疗组患者的mOS2为6.0个月，显著高于未治疗组的1.0个月（*P* < 0.05）；确诊PC后，含有血管生成抑制剂治疗组的mOS2为8.5个月，显著高于其他组（*P* < 0.05）。

**结论:**

肺腺癌和胸腔积液患者或许是发生PC的高危人群；含有血管生成抑制剂治疗策略或许能给PC患者带来更多获益，需要前瞻性研究进一步验证。

## Abstract (en)

**Background and objective:**

Peritoneal carcinomatosis is a rare clinical event in lung cancer and the prognosis is very poor. There are limited data on what factors predict peritoneal progression and affect the outcome. The aim of this study is to investigate investigate the factors associated with peritoneal carcinomatosis.

**Methods:**

The patients with non-small cell lung cancer (NSCLC) from the Department of Medical Oncology and Radiation Sickness, Peking University Third Hospital were eligible for retrospective analysis between August 2010 and August 2018. Clinical factors such as age, gender, histology, pleural effusion and gene mutations with epidermal growth factor receptor/anaplastic lymphoma kinase/ROS proto-oncogene 1 receptor tyrosine kinase (*EGFR*/*ALK*/*ROS1*) were analyzed. Overall survival (OS) was calculated by the *Kaplan-Meier* method.

**Results:**

1.44% (12/836) patients in this study developed peritoneal carcinomatosis and 12 patients with adenocarcinoma had metachronous NSCLC diagnosis and PC. Malignant pleural effusion rates at baseline and at PC diagnosis were separately 50% (6/12) and 100.0% (12/12). Among the 12 patients, 9 patients harbored *EGFR*/*ALK*/*ROS1* mutation. The outcome of patients with *EGFR*/*ALK*/*ROS1* mutation was significantly better than that of patients without *EGFR*/*ALK*/*ROS1* mutation, the mOS1 and mOS2 were separately 26.0 months and 6.0 months versus 10.0 months and 1.5 months (*P* < 0.05). The mOS2 of patients with aggressive treatment after PC diagnosis was 6.0 months, significantly better than 1.0 month of patients with best supportive care (*P* < 0.05). The mOS2 of the patients with angiogenesis inhibitors based-treatment after PC diagnosis was 8.5 months, significantly longer than that of patients with other treatments (*P* < 0.05).

**Conclusion:**

Adenocarcinoma and malignant pleural effusion are highly associated with peritoneal carcinomatosis in patients with advanced NSCLC. Aggressive treatment for lung cancer with PC is encouraged when possible. More patients with PC may benefit from the treatment strategies with angiogenesis inhibitors. Further prospective trials are urgently needed.

肺癌目前是世界范围内发病率和死亡率最高的恶性肿瘤^[1]^，其中80%为非小细胞肺癌（non-small cell lung cancer, NSCLC），约75%的NSCLC确诊时属中晚期^[2, 3]^。靶向和免疫治疗提高了NSCLC患者的无疾病进展生存期（progression-free survival, PFS）和总生存期（overall survival, OS）^[4-6]^，但远处转移仍是NSCLC治疗失败的主要原因，是影响患者预后的重要因素。国内外对NSCLC常见转移（脑、骨、肝和肾上腺）已建立了相应的诊疗指南，但对于少见部位的转移，由于缺乏系统认识，治疗上仍面临巨大挑战，腹膜转移（peritoneal carcinomatosis, PC）就是其中之一。

PC是NSCLC罕见转移之一，由于缺乏特异性症状，早期识别非常困难，尽管早年尸检结果显示PC发生率为2.7%-16%^[7-9]^，但近年来大样本研究发现PC发生率低于5%^[8, 10-12]^。NSCLC的PC患者预后较差，自确诊PC之日起，中位生存期（median overall survival, mOS）不足3个月^[8, 12]^。因此，早期发现及规范治疗是管理PC的关键，但国内外尚缺乏此方面研究。

本研究回顾性分析了2010年8月-2018年8月在北京大学第三医院肿瘤化疗与放射病科确诊且随访资料完整的12例PC患者，初步探讨了患者临床、组织病理和分子病理特征以及治疗等因素对预后的影响。

## 1 对象与方法

### 1.1 研究对象

收集2010年8月-2018年8月在北京大学第三医院肿瘤化疗与放射病科接受治疗的NSCLC患者，通过电子病历信息系统查询诊断中含有“腹腔积液”、“腹膜转移”的患者，同时满足如下标准。纳入标准：组织或细胞病理证实为NSCLC；影像学资料完整；具备PC影像典型特征[计算机断层扫描（computed tomography, CT）或磁共振成像（magnetic resonance imaging, MRI）提示“腹膜增厚、腹膜结节”、“网膜饼”、“肠系膜增厚及结节”、“腹水]^[13]^或细胞学证实为PC。排除标准：有胃癌、结直肠癌或卵巢癌病史；影像学特征缺乏且无细胞学结果。筛查NSCLC患者836例，共14例患者诊断PC，因缺乏影像特征且无细胞学诊断剔除2例，最终12例患者入组本研究。

### 1.2 资料收集

采集并记录患者确诊NSCLC时的临床资料，包括年龄、性别、肿瘤分期、病理类型、基因[表皮生长因子受体（epidermal growth factor receptor, EGFR）/间变性淋巴瘤激酶（anaplastic lymphoma kinase, ALK）/ROS原癌基因1受体酪氨酸激酶（ROS proto-oncogene 1 receptor tyrosine kinase, ROS1）]结果、有无胸腔积液及肿瘤治疗（包括化疗、靶向治疗、抗血管治疗、免疫治疗等）、治疗疗效和生存状态。

### 1.3 评估标准及观察指标

肿瘤分期依据国际肺癌研究协会颁布的第7版分期标准^[14]^。本研究主要观察mOS1（median overall survival 1）和mOS2（median overall survival 2）。mOS1是指从确诊不可切除的中晚期肺癌开始或根治性切除术后发生复发和/或转移开始至死亡或随访终点的时间，mOS2是指从确诊PC开始至死亡或随访终点的时间。

### 1.4 随访

通过定期来院或电话随访，随访开始时间为2010年8月，末次随访时间为2019年1月，随访完成率100.0%。

### 1.5 统计学方法

应用SPSS 19.0统计学软件进行分析。采用*Kaplan-Meier*方法进行生存分析，*Log-rank*检验差异性。全部统计检验均为双侧概率检验，检验水准*α*=0.05，以*P* < 0.05为差异有统计学意义。

## 2 结果

### 2.1 PC患者临床特征

共12例PC患者入组本研究，均为异时性转移，其中细胞学和影像诊断5例（图 1），仅影像诊断7例。PC发生率为1.44%（12/836）。12例PC患者，中位年龄47.5岁（36岁-75岁）， < 60岁的患者居多，占75.0%（9/12）；女性患者偏高，占66.7%（8/12）；12例患者病理均为腺癌，含*EGFR*突变7例（外显子19缺失4例，外显子21 L858R点突变2例，外显子20插入突变1例），含有*ROS1*突变2例，无*EGFR*/*ALK*/*ROS1*突变3例；12例患者在确诊肺癌时，合并胸腔积液6例，腹盆腔脏器转移2例和腹膜后淋巴结转移1例；在确诊PC时，合并胸腔积液12例，腹盆腔脏器转移7例，腹膜后淋巴结转移6例（表 1）。

**1:**
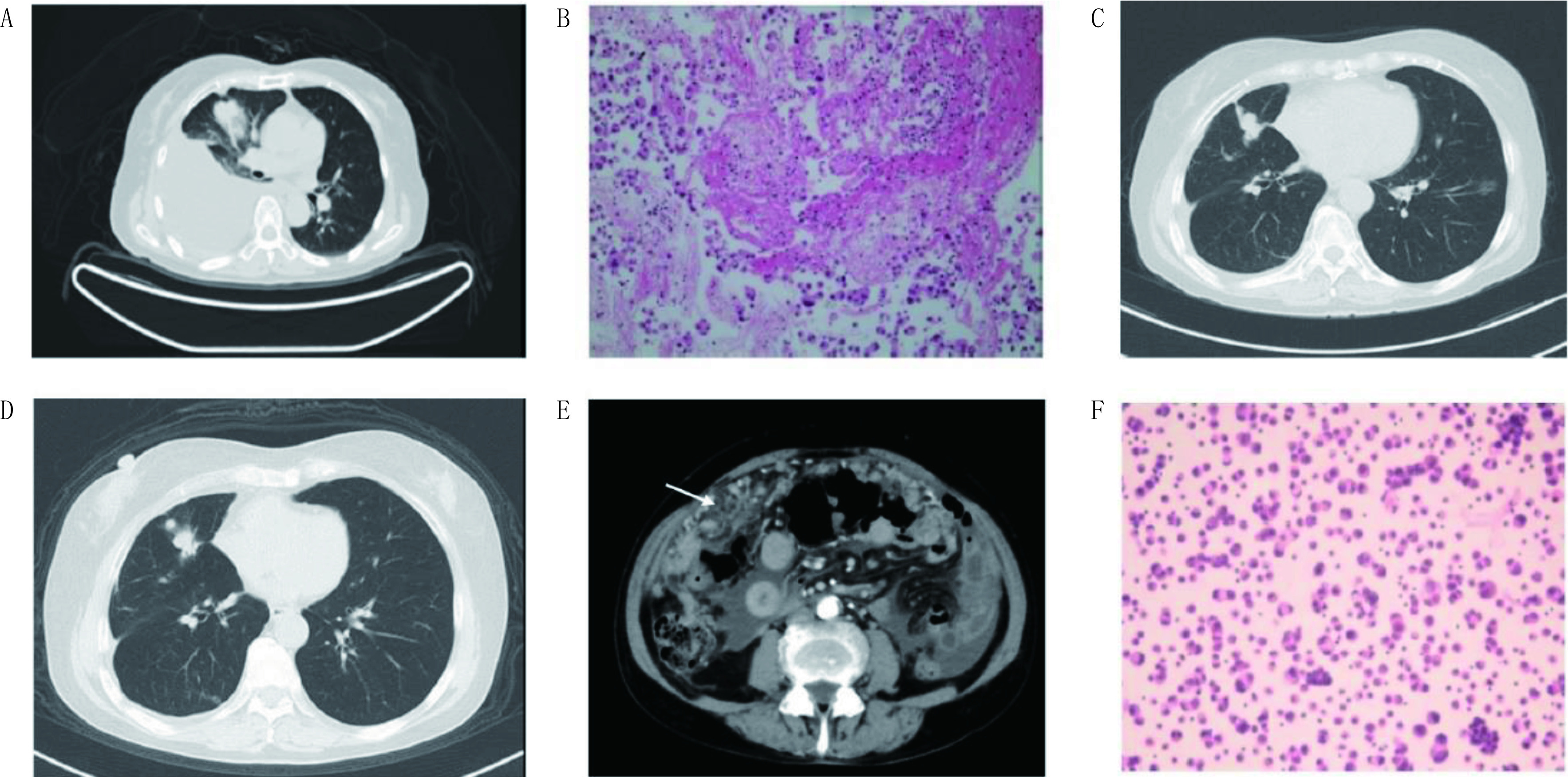
1例*EGFR*突变PC患者的影像及细胞学表现。A：确诊非小细胞肺癌时；B：确诊时胸水中找到腺癌细胞（HE, ×100）；C：吉非替尼治疗4个月；D：吉非替尼治疗8个月；E：吉非替尼治疗8个月（发生腹膜转移，箭头提示腹膜结节，腹膜增厚）；F：腹水中找到腺癌细胞（HE, ×100）。 The radiology and cytology of one PC patient with *EGFR* mutation. A: at NSCLC diagnosis; B: Cytology of pleural effusion at diagnosis (HE, ×100); C: 4 months after gefitinib; D: 8 months after gefitinib; E: 8 months after gefitinib (at PC diagnosis, white arrow-peritoneal carcinomatosis); F: Cytology of ascites (HE, ×100).

**1:** 12例腹膜转移非小细胞肺癌临床病理特征 The clinical characteristics of 12 non-small cell lung cancer patients with peritoneal carcinomatosis (PC)

Characteristics	Peritoneal metastasis [*n*/*N*(%)]
Gender	12/12 (100.0)
Male	4/12 (33.3)
Female	8/12 (66.7)
Age (yr)	12/12 (100.0)
Median	47.5 (36.0-75.0)
≥60	3/12 (25.0)
< 60	9/12 (75.0)
Stage	12/12 (100.0)
Ⅱ	2/12 (16.7)
Ⅲ	1/12 (8.3)
Ⅳ	9/12 (75.0)
Pathology	12/12 (100.0)
Adenocarcinoma	12/12 (100.0)
Diagnosis of PC	12/12 (100.0)
Cytological and radiological	5/12 (41.7)
Radiological	7/12 (58.3)
*EGFR*/*ALK*/*ROS1* mutation status	12/12 (100.0)
No	3/12 (25.0)
Yes	9/12 (75.0)
*EGFR* mutation	7/12 (58.3)
19del	4/12 (33.3)
21L858R	2/12 (16.7)
Other	1/12 (8.3)
*ROS1* mutation	2/12(16.7)
Metastasis at NSCLC diagnosis	12/12 (100)
With pleural effusion	6/12 (50.0)
Without pleural effusion	6/12 (50.0)
With abdominal and/or pelvic viscera metastasis	2/12 (16.7)
Without abdominal and/or pelvic viscera metastasis	10/12 (83.3)
With retroperitoneal lymph node metastasis	1/12 (8.3)
Without retroperitoneal lymph node metastasis	11/12 (91.7)
Metastasis at PC diagnosis	12/12 (100.0)
With pleural effusion	12/12 (100.0)
Without pleural effusion	0/12 (0.0)
With abdominal and/or pelvic viscera metastasis	7/12 (58.3)
Without abdominal and/or pelvic viscera metastasis	5/12 (41.7)
With retroperitoneal lymph node metastasis	6/12 (50.0)
Without retroperitoneal lymph node metastasis	6/12 (50.0)
EGFR: epidermal growth factor receptor; ALK: anaplasticlymphoma kinase; ROS1: receptor tyrosine kinase 1; NSCLC: non-small cell lung cancer.	

### 2.2 PC患者治疗情况

确诊NSCLC后，7例*EGFR*突变患者均接受了表皮生长因子受体酪氨酸激酶抑制剂（epidermal growth factor receptor tyrosine kinase inhibitors, EGFR TKIs）治疗，一线治疗5例，二线治疗2例，耐药后4例患者接受了二次基因检测，1例发生*EGFR* T790M突变，后线给予了AZD9291治疗；2例ROS1患者接受了克唑替尼治疗，1例克唑替尼治疗失败后接受了Lorlatinib治疗；共有6例一线接受了化疗（表 2）。确诊PC后，9例患者接受了治疗，仅接受化疗或TKIs治疗者各2例，联合血管生成抑制剂（包括阿帕替尼、安罗替尼、贝伐珠单抗或重组人血管内皮抑制素）治疗的5例；3例患者因体力状况未再接受治疗（表 2）。图 2显示的是1例吉非替尼治疗失败后发生PC的患者，吉非替尼联合安罗替尼治疗1个月和3个月后腹水减少，疾病得到控制达6个月。

**2:**
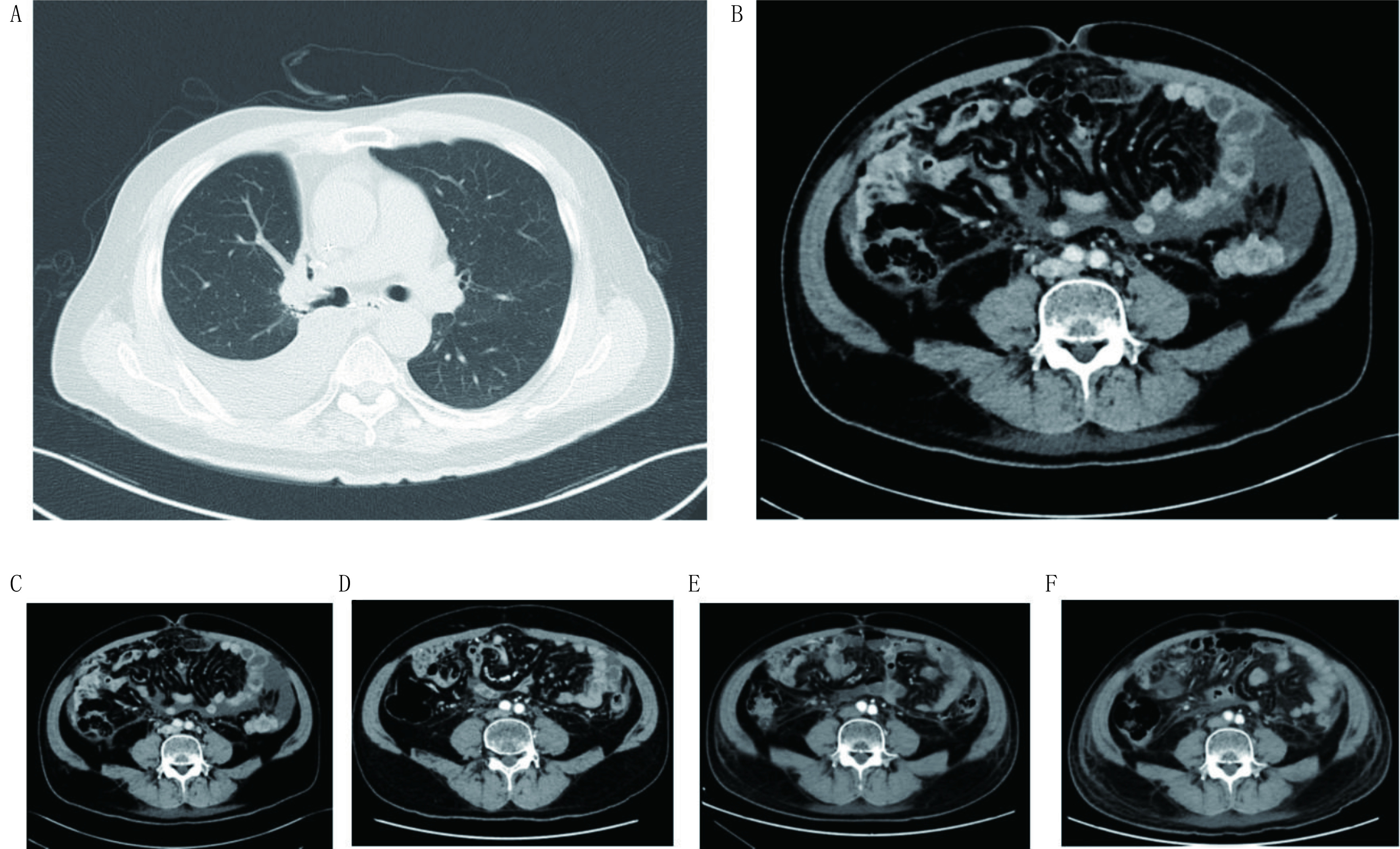
吉非替尼联合安罗替尼治疗患者1例。A：确诊PC胸部CT；B：确诊PC时腹部CT；C：确诊PC时；D：吉非替尼联合安罗替尼治疗1个月（腹水减少）；E：吉非替尼联合安罗替尼治疗3个月；F：确诊PC 6个月。 One patient with treatment of gefitinib and anlotnib. A: Chest CT image at PC diagnosis; B: Abdomen CT image at PC diagnosis; C: at PC diagnosis; D: 1 month after gefitinib plus anlotinib (only minimal ascites); E: 3 months after gefitinib plus anlotinib; F: 6 months after PC diagnosis.

**2:** 12例PC非小细胞肺癌患者治疗情况及预后 The treatment strategies, responses and outcomes of the 12 NSCLC patients with PC

	Age/gender	*EGFR*/*ROS1*/*ALK* mutation	Resistance mutation	First line treatment	Treatment after PC diagnosis	OS after PC diagnosis (month)	Overall survival (month)
Patient 1	63/M	*EGFR* 21L858R	Without T790M	Gefitinib	Icotinib plus Apatinib/bevacizumab plus PP/Doc/Doc plus Nedaplatin/Anlotinib	12.5	26.0
Patient 2	49/M	*EGFR* 19del	Without T790M	PP	Nivolumab/gefitinib plus Anlotinib/Afatinib plus Anlotinib	6.0	31.0
Patient 3	75/F	*EGFR* 19del	-	Gefitinib	Docetaxel	NR	NR
Patient 4	36/F	*ROS1*	-	PP	Crizotinib/Lorlatinib	8.5	38.0
Patient 5	53/M	*EGFR* 20 Insert	-	PP	Cisplatin plus bevacizumab/erlotinib	2.2	9.5
Patient 6	65/F	*EGFR* 21L858R	Without T790M	Gefitinib	Bevacizumab/endostar/gemcitabine	5.0	36.0
Patient 7	45/F	No	-	PP	Erlotinib	1.0	3.0
Patient 8	47/F	*EGFR* 19del	-	Gefitinib	Docetaxel	6.5	18.0
Patient 9	37/M	No	-	PP	No treatment	2.0	10.0
Patient 10	48/F	*EGFR* 19del	With T790M	Gefitinib	AZD9291/bevacizumab	8.5	18.0
Patient 11	47/F	No	No	Docetaxel plus Carboplatin	No treatment	1.5	14.0
Patient 12	42/F	*ROS1*	-	Crizotinib	No treatment	1.0	3.0
PP: pemetrexed plus cisplatin, NR: not reached; OS: overall survival.							

### 2.3 PC患者生存情况

12例PC患者中11例发生了死亡，mOS1 18.0个月（95%CI: 3.0-38.0），mOS2 5.0个月（95%CI: 1.0-12.5）。确诊NSCLC时，合并胸腔积液患者的mOS2和mOS1劣于无胸腔积液者，mOS2分别为3.0个月（95%CI: 1.0-5.1）和6.0个月（95%CI: 0.0-12.9），*P*=0.280（图 3A）；mOS1分别为18.0个月（95%CI: 8.7-27.3）和26.0个月（95%CI: 9.3-42.7），*P*=0.238（图 3C）；但未见显著差异；含有*EGFR*/*ROS1*突变患者的mOS2和mOS1显著高于无突变者，mOS2分别为6.0个月（3.9-8.1）和1.5个月（95%CI: 0.7-2.0），*P*=0.000，（图 3B）。mOS1分别为26.0个月（95%CI: 9.4-42.6）和10.0个月（95%CI: 0.0-21.2），*P*=0.006（图 3D）；确诊PC后，接受治疗组的mOS2显著高于未治疗组，分别为6.0个月（95%CI: 3.1-8.9）和1.0个月（95%CI:.-.），*P*=0.006（图 3E）；含有血管抑制剂治疗组、无血管抑制剂治疗组和未治疗组的mOS2 8.5个月（95%CI: 1.0-16.0）、3.0个月（95%CI: 0.0-7.4）和1.0个月（95%CI:.-.），*P*=0.009（图 3F）。

**3:**
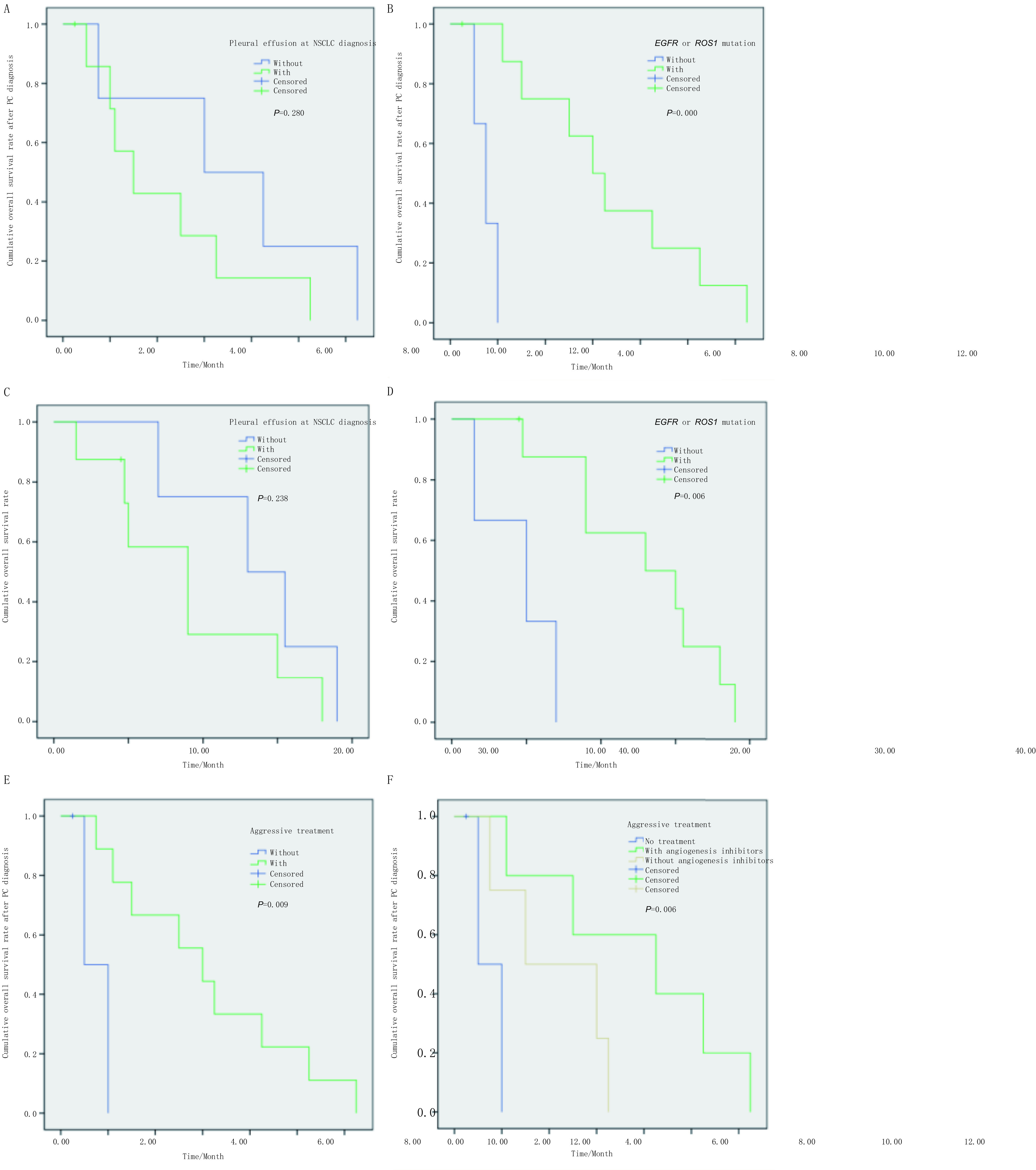
患者累积生存率曲线。A：有无胸水对确诊PC后患者生存的影响；B：基因突变状态对确诊PC后生存的影响；C：有无胸水对患者总生存的影响；D：基因突变状态对确诊PC后总生存的提高；E：治疗与否对确诊PC后患者生存的影响；F：治疗类型对确诊PC后患者生存的影响。 The curve of cumulative overall survival rate. A: The OS after PC diagnosis of patients with/without pleural effusion; B: The OS after PC diagnosis of patients with/without *EGFR*/*ROS1* mutation; C: The OS of patients with/without pleural effusion; D: The OS of patients with/without *EGFR*/*ROS1* mutation; E: The OS after PC diagnosis of patients with/without treatment; F: The OS after PC diagnosis of patients with different treatment groups.

## 3 讨论

PC是NSCLC罕见转移部位之一，由于缺乏特异性症状，早期发现PC困难。本研究中PC发生率为1.44%（12/836），与国内曾军等^[15]^报道的1.30%（10/767），Hsu等^[16]^报道的1.13%（3/265）以及Satoh等^[8]^报道的1.2%(12/1, 041）类似，高于Niu等^[10]^报道的0.84%（24/2, 872），但低于Patil等^[11]^报道的8%（33/410）。各组发生率差异较大在于研究人群不同，如本研究中*EGFR*/*ROS1*突变患者居多，占75%（9/12），且50%（6/12）患者在确诊NSCLC时合并胸腔积液，与Patil等^[11]^发现的79% PC（26/33）患者合并胸腔积液类似，因此明确PC的高危人群特征显得尤为重要。

本研究中12例PC患者均为肺腺癌，50%合并恶性胸腔积液，确诊PC时100%（12/12）合并胸腔积液，与其他几项研究结果类似。Satoh等^[8]^26年观察的1, 041例肺癌患者中的12例发生PC，病理类型腺癌居多^[8]^；Su等^[12]^报道的30例PC患者中，亦是肺腺癌居多。Patil等^[11]^观察410例NSCLC患者，33例PC患者中26例合并胸腔积液。因此，肺腺癌和胸腔积液的肺癌患者或许是需要关注的PC高危人群。可能因为：①肺腺癌易发生血行转移，而血行转移是NSCLC发生PC的主要机制之一；②肺腺癌含有驱动基因突变的比例高，靶向治疗在提高患者生存期的同时，也延长了肿瘤细胞适应腹膜微环境的时间，进而提升了PC的发生几率；③Takagi等^[17]^在淋巴管平滑肌增多症肺疾病患者中发现，隔膜具有丰富的淋巴网络，乳糜样积液可以通过隔膜病灶发生淋巴管渗透到腹腔，因此伴有恶性胸腔积液的患者，不除外肿瘤侵犯隔膜，进而通过淋巴道转移导致PC。

本研究12例PC患者，自PC确诊后的中位生存期（mOS2）是5.0个月，接受治疗组的mOS2为6.0个月，显著高于未接受治疗组的1.0个月，与既往研究结果相似。Satoh等^[8]^和Su等^[12]^发现未接受治疗的PC患者，mOS2分别为15 d和2个月；Niu等^[10]^报道了24例肺癌PC的患者，mOS2为2.8个月，1年生存率为14.9%。Abbate等^[18]^报道的60例肺癌PC患者，mOS2为3.5个月。综上可见，肺癌PC患者预后差，其原因可能是：①肺癌PC患者多合并腹腔积液，一旦出现恶性腹水，通常意味着患者已进入终末期，大多数患者身体状况差，失去了治疗机会；②按照“种子-土壤”学说而言，腹膜不是肺癌细胞适合生存的微环境，肿瘤细胞需要经历较长时间适应才能存活，一旦存活下来，肿瘤细胞的侵袭和增殖能力将增强，进而加速对机体的破坏。因此，肺癌PC的治疗目前仍是临床上面临的巨大挑战。

本研究12例PC患者中，治疗组9例患者的mOS2为6.0个月，含有血管生成抑制剂治疗组为8.5个月，可见有效的治疗能给PC患者带来生存获益。Su等^[12]^研究中也证实了这一结果，9例接受化疗的PC患者生存率显著提高。Niu等^[10]^在193例罕见转移的肺癌患者中发现，全身治疗联合局部治疗组比仅全身治疗组和仅最佳支持治疗组的生存期显著延长，mOS2分别为12.5个月、7.4个月比3.4个月（*P* < 0.01）。此外，Hsu等^[16]^报道贝伐珠单抗联合化疗或者靶向治疗带来PC患者mOS2延长。上述PC患者获益的原因可能是：①能够接受化疗的患者，身体状态本身就好于不能接受治疗组的患者；②PC患者能对治疗产生应答，特别是血管生成阻断治疗，如血管内皮生长因子（vascular endothelial growth factor, VEGF）阻断，目前已经清楚VEGF过表达能显著增加血管渗透性，诱导胸水或腹水发生^[19]^，Verheul等^[20]^发现VEGF在恶性腹水中的活性水平显著升高，阻断VEGF能够给腹腔积液带来获益。本研究首次报道了安罗替尼或阿帕替尼联合靶向治疗或化疗对PC患者有效，这类药物亦是VEGF受体、纤维母细胞生长因子受体、血小板源性生长因子受体等多个靶点的酪氨酸酶抑制剂，抑制血管生成；③本研究中含有基因突变患者的生存期更长，与Abbate等^[18]^研究结果类似，Kobayashi等^[21]^报道1例腹腔积液检测到EGFR S768I突变的PC患者，应用阿法替尼治疗有效，无疾病进展生存期已超过12个月。因此，对于身体状况较好的PC患者，积极治疗能够给患者带来获益，化疗或靶向联合抗血管生成治疗或许能给PC患者带来更多获益。

本研究的缺陷在于：①样本量较小，存在选择性偏倚可能；②本研究主要依靠细胞学或影像学诊断，未纳入影像特征不典型的患者和因疾病处于终末期未诊治，可能合并PC的患者，因此PC的发生率可能被低估。本研究发现12例PC患者均为腺癌，初诊时合并胸腔积液率高，提示我们腺癌、胸腔积液患者或许是PC的高危人群，需要引起临床重视。靶向治疗及抗血管生成治疗或许是PC治疗的最佳策略，需要前瞻性临床研究验证。
